# A case of extracorporeal carbon dioxide removal for postpneumonectomy acute respiratory distress syndrome

**DOI:** 10.1016/j.xjtc.2024.10.007

**Published:** 2024-10-18

**Authors:** Annette M. Ilg, Ryan M. Gardner, Stephen D. Hallisey, Antonio Coppolino, Raghu R. Seethala

**Affiliations:** a Division of Emergency Critical Care Medicine, Department of Emergency Medicine, Brigham and Women's Hospital, Boston, Mass; b Division of Anesthesia Critical Care, Department of Anesthesia, Tufts Medical Center, Boston, Mass; c Division of Thoracic Surgery, Department of Surgery, Brigham and Women's Hospital, Boston, Mass

**Figure:**
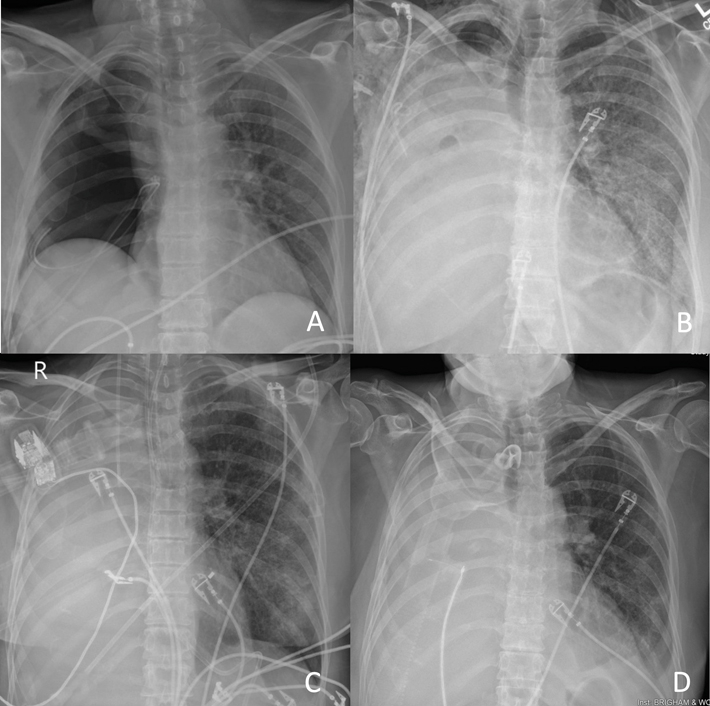
Post-pneumonectomy radiograph evolution. A, POD 0 Right pneumonectomy. B, POD 12 Single-lung ARDS. C, POD 18 Before ECCO2R with refractory hypercapnic respiratory failure despite maximal ventilatory support. D, POD 30 After ECCO2R improving to tracheostomy collar.

**Central Message**

Postpneumonectomy acute respiratory distress syndrome is an uncommon, high-mortality disease. Extracorporeal carbon dioxide removal provides a novel approach to management.

Extracorporeal life support (ECLS) including extracorporeal carbon dioxide removal (ECCO_2_R) and venovenous (VV) extracorporeal membrane oxygenation (ECMO) provide alternative strategies in refractory respiratory failure. ECCO_2_R enables carbon dioxide (CO_2_) removal through a VV circuit with a mechanical pump or an arterial-venous system reliant on native cardiac function.^1^ ECLS can help minimize ventilator-induced lung injury (VILI), correct gas exchange abnormalities, and facilitate lung recovery.^1^ To date, ECCO_2_R has been used in acute respiratory distress syndrome (ARDS), asthma and chronic obstructive pulmonary disease exacerbations, severe pneumonia, and as a bridge to lung transplant.

Postpneumonectomy ARDS is a rare disease with high mortality (40%-60%),^2^^,^^3^ and no society guidelines exist. We describe the first case of postpneumonectomy ARDS supported with the Hemolung Respiratory Assist System (Hemolung RAS; ALung Technologies) since it was approved by the Food and Drug Administration.^3^, ^4^, ^5^ Institutional review board approval was not required; the patient provided informed written consent for publication.

## Case Report

A 53-year-old female patient with chronic obstructive pulmonary disease and lung squamous cell carcinoma status-post chemotherapy and radiation underwent right robotic converted to open carinal pneumonectomy with positive margin. She was extubated postoperatively and recovered well through postoperative day (POD) 10.

On POD 11, she developed hypoxic respiratory failure requiring nonrebreather. Despite diuresis and antibiotics, she required bronchoscopic-guided intubation. She was supported on pressure-controlled ventilation (pressure control [PC] 14 cm H_2_O, tidal volume 190 mL, 4 mL/kg, respiratory rate [RR] 32 breaths/min, minute ventilation 6.1 L/min, plateau pressure [Pplat] 24 cm H_2_O). Her arterial blood gas demonstrated pH 7.20, arterial carbon dioxide tension (Paco_2_) 84 mm Hg, and arterial oxygen tension 89 mm Hg. Her bronchoalveolar lavage and computed tomography angiography scan were negative for infectious or thromboembolic etiologies, with findings consistent with nonspecific ARDS. Accordingly, she was paralyzed for several days with P/F (ratio of arterial oxygen partial pressure [PaO2 in mmHg] to fractional inspired oxygen [FiO2 expressed as a fraction not a percentage]) less than 150 with ongoing diuresis and antimicrobials.

On day 6 of ventilation, her hypercarbia worsened, prompting increased ventilatory settings (RR 36, PC 12 cmH_2_O, Pplat 24 cmH_2_O), without improvement observed. Overnight PC was increased to 18 cmH_2_O, again without improvement observed. The following morning, PC was further increased to 22 cmH_2_O and RR decreased to 32 (tidal volume 200 mL, 4.2 mL/kg, minute ventilation 6.4 L/min, driving pressure 22 cmH_2_O, positive end-expiratory pressure 11 mH_2_O, Pplat 33 cmH_2_O). Her hypoxemia had improved (P/F (ratio of arterial oxygen partial pressure [PaO2 in mmHg] to fractional inspired oxygen [FiO2 expressed as a fraction not a percentage]) 162), although her hypercarbia remained refractory (Paco_2_ >101 mm Hg, pH 7.27).

After a multidisciplinary ECLS discussion, ECCO_2_R was deployed as bridge to recovery on POD 19. She was cannulated through her femoral vein with the Hemolung RAS via a proprietary 15.5-French dual lumen catheter. ECCO_2_R was initiated (speed 1400 rpm, blood flow rate 520 mL/min, CO_2_ removal 96 mL/min, sweep 10 L/min) on therapeutic heparin. Her ventilator was weaned later that day to PC 15 cmH_2_O and RR 24.

After 48 hours, her pH and Paco_2_ were 7.48 and 59 mm Hg, and her ventilator was further weaned (PC 12 cmH_2_O, RR 16). She continued to improve with ongoing diuresis and underwent percutaneous tracheostomy POD 22, ECCO_2_R day 3.

**Figure 1:**
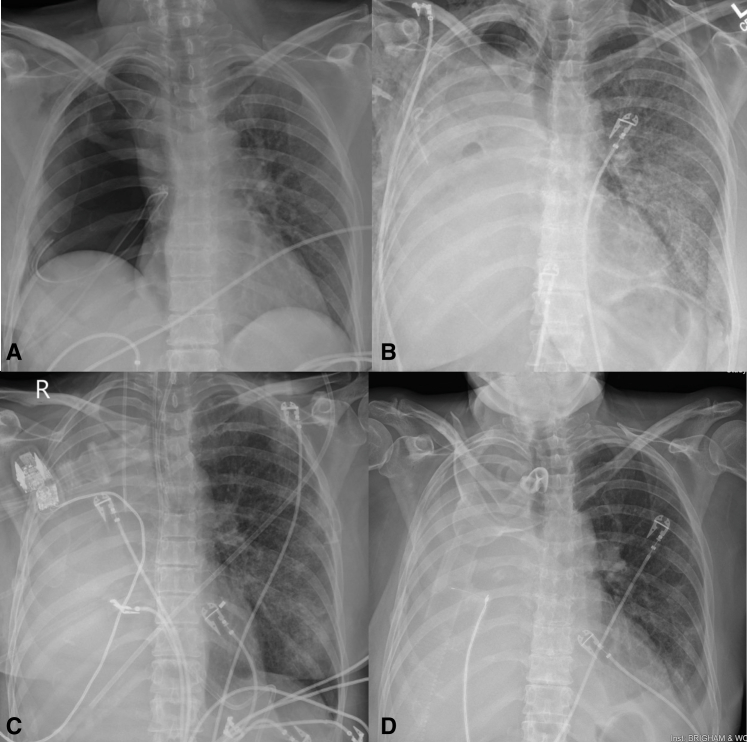
Postpneumonectomy radiograph evolution. A, POD 0 Right pneumonectomy. B, POD 12 single-lung ARDS. C, POD 18 before ECCO_2_R with refractory hypercapnic respiratory failure despite maximal ventilatory support. D, POD 30 after ECCO_2_R improving to tracheostomy collar. *POD*, Postoperative day; *ARDS*, acute respiratory distress syndrome; *ECCO2R*, extracorporeal carbon dioxide removal.

Over ECCO_2_R days 4 to 7, her sweep was weaned to 4 L/min with stable ventilation. After 8 days of extracorporeal support, her sweep was weaned off, and she was decannulated on ECCO_2_R day 9 with arterial blood gas pH 7.46, Paco_2_ 47 mm Hg, and arterial oxygen tension 113 mm Hg. No ECCO_2_R-related complications were experienced. Over the next 72 hours, she weaned to heated tracheostomy collar and transferred to a step-down unit. Radiographs of the patient's chest throughout her postoperative course are included in Figure 1.

The patient continued to recover and was discharged POD 51 left the hospital to a rehabilitation facility. A few months after hospital discharge, she had metastatic recurrence treated with pembrolizumab and radiation therapy over the following year.

## Discussion

This case demonstrates a novel approach to management in postpneumonectomy ARDS, a high-mortality disease with limited targeted studies. Previous ARDS trials have demonstrated that high Pplat (>30 cmH_2_O) and driving pressures (>15 cmH_2_O)^E1^ are associated with VILI and increased mortality. Thus, formal ARDS guidelines recommend low-tidal volume ventilation (<6 mL/kg), conservative fluid balance, prone positioning, positive end-expiratory pressure optimization, and consideration of ECLS if severe (P/F (ratio of arterial oxygen partial pressure [PaO2 in mmHg] to fractional inspired oxygen [FiO2 expressed as a fraction not a percentage]) < 80).^E1^

However, patients with ARDS postpneumonectomy have several key differences: increased pulmonary vascular resistance, heightened inflammatory response, ischemia-reperfusion injury, and positive perioperative fluid balance.^E2^ Moreover, patients who undergo pneumonectomy postoperatively experience pain, splinting, and atelectasis, further compromising respiratory mechanics. All these factors predispose to reduced compliance of the remaining lung with a bronchial stump at risk of dehiscence. Thus, traditional ARDS targets may actually result in poor lung protection, and proning postsurgical patients has notable risks. Given the potential VILI, novel support strategies and targeted trials are needed to guide management.

In the absence of randomized controlled trials and guidelines in patients postpneumonectomy, collaborative discussion on how to think through this problem becomes paramount. Patients with single lungs are unique and ultra-lung protective ventilation (4 mL/kg) with more conservative plateau and driving pressures should be considered early. For patients with refractory ARDS, ECCO_2_R and VV-ECMO may offer benefit earlier in management than traditional targets.

VV-ECCO_2_R primarily provides support for hypercarbic respiratory failure and has several advantages: single-site cannulation, smaller cannula size, increased patient mobility, and lower blood flow rates with less resource-intensive initiation and maintenance. Specifically, VV- ECCO_2_R can be initiated at bedside without fluoroscopy or transesophageal echocardiography guidance and requires fewer specialized staff for monitoring. The primary disadvantages of VV- ECCO_2_R are that oxygenation support is negligible and full anticoagulation is required.

In contrast, the advantages of VV-ECMO include comprehensive support for both hypercarbic and hypoxic respiratory failure and greater flow rates, allowing anticoagulation levels to be titrated to patient risk profile. With proper securement of cannulae, patients can be mobilized pending institutional policy and provider experience. The disadvantages of VV ECMO are that it requires more resources for initiation (potentially transesophageal echocardiography or fluoroscopy) and monitoring (dedicated specialized staff) of the circuit and oxygenator function. Whether 2-site or a single-site dual-lumen strategy, VV ECMO is susceptible to recirculation requiring further resources to reposition. Depending upon the patient's type of respiratory failure (hypoxic, hypercarbic, mixed), risk profile for bleeding, and need to mobilize in the context of hospital policy, the appropriate support platform can be selected.

## Conclusions

As the first case since approval by the Food and Drug Administration of the Hemolung RAS in postpneumonectomy ARDS, this highlights the opportunity to allow safe lung recovery through extrapulmonary support through one small dual-lumen catheter placed at bedside. Further studies are needed in postpneumonectomy ARDS, as no guidelines exist.

## Conflict of Interest Statement

Dr Seethala has received consulting fees from LivaNova not related to this work. The other authors reported no conflicts of interest.

The *Journal* policy requires editors and reviewers to disclose conflicts of interest and to decline handling or reviewing manuscripts for which they may have a conflict of interest. The editors and reviewers of this article have no conflicts of interest.
